# Non-operative management of gallstone sigmoid ileus in a patient with a prostatic cancer

**DOI:** 10.1093/jscr/rjad331

**Published:** 2023-06-09

**Authors:** Ahmed M AlMuhsin, Abdulaziz Bazuhair, Omar AlKhlaiwy, Rami O Abu Hajar, Thabit Alotaibi

**Affiliations:** Department of General Surgery, Security Forces Hospital, Dammam 31413, Saudi Arabia; Department of General Surgery, Security Forces Hospital, Dammam 31413, Saudi Arabia; Department of General Surgery, Security Forces Hospital, Dammam 31413, Saudi Arabia; Department of General Surgery, Security Forces Hospital, Dammam 31413, Saudi Arabia; Adult Critical Care Department, King Fahd University Hospital, Imam Abdulrahman Bin Faisal University, Dammam 32314, Saudi Arabia

## Abstract

Gallstone ileus is an uncommon complication of calculus cholecystitis through the formation of a biliary enteric fistula. The risk of mechanical obstruction caused by gallstones is increased with its size, in addition to chronic constipation, neoplasm and diverticulitis, to name a few. Here, we present a case of an 89-year-old male patient who presented with signs of bowel obstruction, which was found to be a gallstone impacted in the sigmoid colon. Considering the patient’s stable condition and his comorbidities, a conservative approach was opted including IV fluids, fleet enema and bowel rest. Colonoscopy was performed and confirmed the passage of the stone. With no consensus regarding the management, the literature emphasizes a tailored approach to each case considering all possible operative and non-operative approaches. Some reports show promising results with non operative management. Gallstone ileus remains a challenging case, and further studies for the best treatment modalities are needed.

## INTRODUCTION

Gallstone ileus occurs when a gallstone causes a blockage in the intestine due to the formation of a biliary enteric fistula. This is a rare complication of calcular cholecystitis [1]. In the USA, the incidence of gallstones is up to 15% among adults [2], and approximately 0.3–0.5% of patients with gallstones will develop gallstone ileus [3]. Computed tomography (CT) is the preferred imaging modality for the diagnosis of gallstone ileus, with high sensitivity [4]. It can also detect other stones in the gastrointestinal tract in up to 12.5% of cases [4], evidence of intestinal obstruction, pneumobilia, air in the gallbladder and, occasionally, the presence of the biliary enteric fistula [5]. Commonly, the location of the fistula is between the gallbladder and duodenum [1]. In a review by Reisner and Cohen, the terminal ileum was the most common site of stone impaction in 60.5% of cases, whereas the colon was only in 4.1% of cases [6]. The literature reports that gallstones as small as 2.3 cm have been found in the sigmoid colon, as noted by Cunha *et al.* [7]. Inukai *et al.* have reported the largest gallstone on record, measuring 7 cm and causing obstruction in the sigmoid colon [8]. Here we present a case report and a literature review of a gallstone sigmoid ileus in a patient with prostatic cancer, causing narrowing of the rectosigmoid junction.

## CASE REPORT

**Figure 1:**
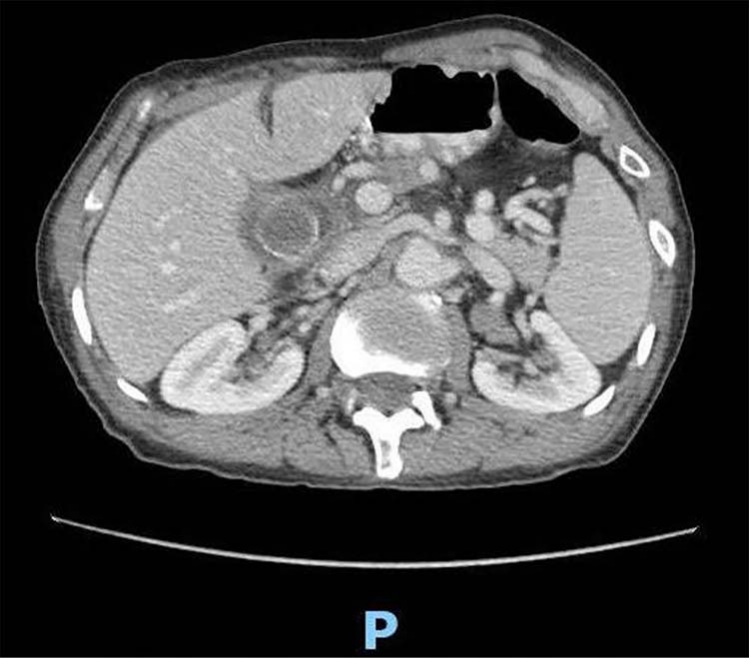
Enhanced CT scan of the abdomen confirming the presence of a 2.5-cm gallstone.

A 89-year-old male patient was admitted to the emergency department complaining of generalized abdominal pain for 4 days. The pain was associated with repeated vomiting and obstipation. He also reported a history of chronic constipation. However, he denied any history of melena, hematochezia, weight loss, night sweats or fever. His past medical history revealed a history of prostatic cancer and pulmonary fibrosis caused by tuberculosis. The patient was diagnosed with symptomatic gallstones two years prior to his presentation, after undergoing an enhanced CT scan of the abdomen (Fig. 1) for abdominal pain. Although he was offered cholecystectomy, he refused the surgery due to his high-risk medical condition.

**Figure 2:**
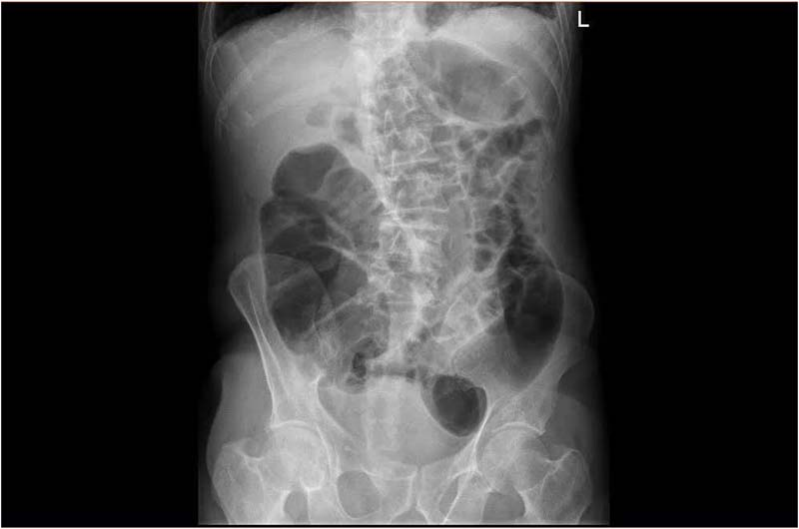
Plain abdominal X ray showing a dilated large bowel loop.

**Figure 3:**
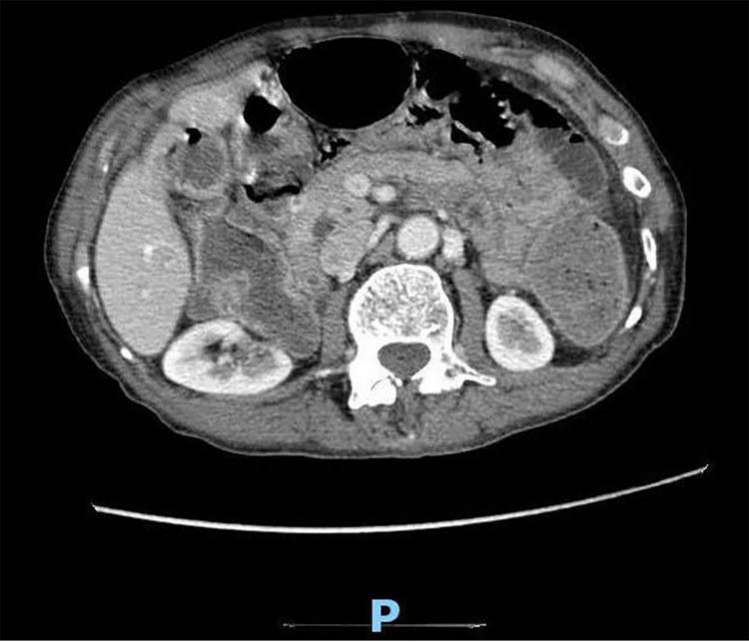
Enhanced CT scan of the abdomen showing evidence of partial large bowel with pneumobilia, gallbladder seen with interrupted wall and suspected fistula with the hepatic flexure (axial view).

**Figure 4:**
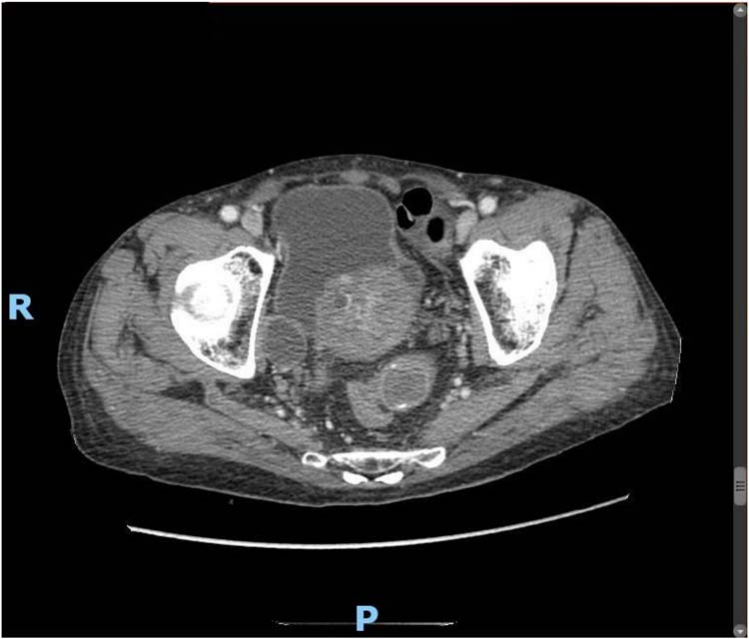
Enhanced CT scan of the abdomen showing a gallbladder stone impacted at the sigmoid colon, with a significantly enlarged prostate causing narrowing of the rectosigmoid junction (axial view).

**Figure 5:**
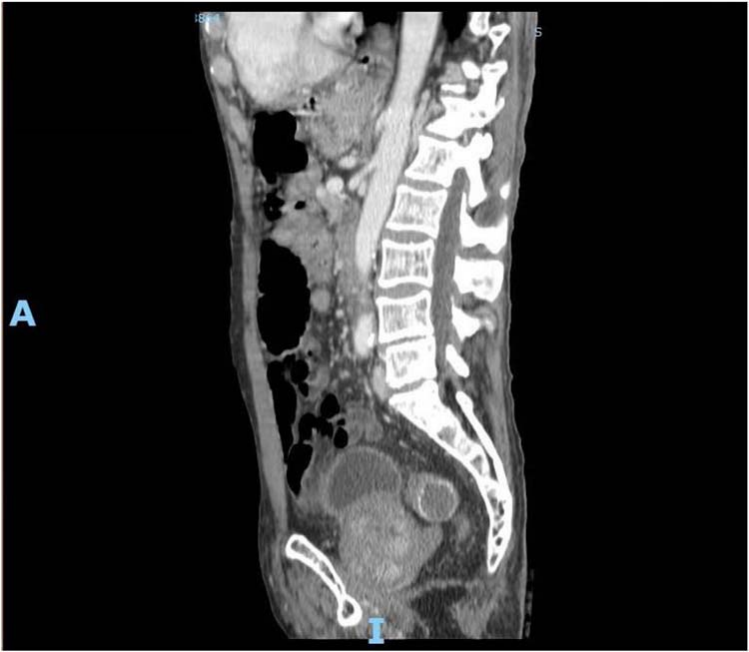
Enhanced CT scan of the abdomen showing a gallbladder stone impacted at the sigmoid colon, with a significantly enlarged prostate causing narrowing of the rectosigmoid junction (sagittal view).

Upon clinical examination, the patient was vitally stable. Abdominal examination revealed a distended abdomen with generalized tenderness. Digital rectal examination revealed an empty rectum with no palpable masses. Laboratory investigations were unremarkable (Table 1). Abdominal X-ray was done (Fig. 2) and showed dilated large bowel loops suggestive of bowel obstruction. Enhanced CT scan of the abdomen and pelvis was obtained (Figs 3–5) and showed a dilated large bowel loop, with air fluid level. A 2.5-cm gallstone was noted, impacting the sigmoid colon and causing partial large bowel obstruction. There was evidence of pneumobilia with air foci within the gallbladder with a suspected fistula with the hepatic flexure.

**Table 1:** The patient’s laboratory investigations

**Labs**	**Results**	**Normal range**
WBC	5.70 x10e3/uL	4.00–11.00
Hemoglobin	13.0 g/dL	13.5–17.2
Platelets	316 x10e3/uL	150–450
Potassium	3.80 mmol/L	3.60–5.00
Sodium	135 mmol/L	136.0–145.0
Chloride	100 mmol/L	95.0–110.0
ALT	12 U/L	0.0–55.0
AST	16 U/L	5.00–34.00
GGT	13 U/L	12.00–64.00
ALK	68 U/L	40.0–150.0
Total bilirubin	0.67 mg/dl	0.20–1.20
Direct bilirubin	0.34 mg/dl	<0.5
Lactate	12.1 mg/dL	4.5–19.8
Amylase	64.00 U/L	20.00–160.00

**WBC** = white blood cells, **ALT** = alanine aminotransferase, **AST** = aspartate aminotransferase, **GGT** = gamma-glutamyl transferase, **ALK** = alkaline phosphatase

Due to the patient’s stable clinical condition, a trial of conservative management was attempted. The patient was kept NPO on intravenous fluids, and multiple fleet enemas were given. He passed a moderate amount of stool with some improvement in his overall clinical condition. However, there was no evidence of stone passage. A decision was made to perform a colonoscopy to confirm the passage of the stone and to relieve the obstruction. During the colonoscopy, there were no obstructing lesions or stones identified up to the splenic flexure. The patient was recovering gradually. He had normal bowel motion, a diet was introduced gradually and the patient tolerated oral feeding well. Taking into consideration the patient’s age and his comorbidities, the decision was made not to treat the fistula for the time being. The patient was discharged in a good condition after 3 days of admission. He was followed in the outpatient clinic for 1 year, with no evidence of recurrence or residual symptoms.

## DISCUSSION

Sigmoid gallstone ileus is a rare cause of mechanical bowel obstruction. It occurs when a gallstone passes through a cholecystoenteric or cholecystocolonic fistula [1]. This fistula tract is usually the result of prolonged and substantial inflammation affecting the gallbladder and the biliary tree, eventually leading to adhesion between the gallbladder and the adjacent bowel [8]. Taking into consideration that gallstones are more common in females, and, therefore, they are more likely to experience its complications, and the fact that the prevalence of comorbidities increase with age, it is understandable that the incidence of sigmoid gallstone ileus is higher in females, especially elderly with multiple comorbidities [9].

The risk of stone impaction in the colon is increased by the size of the gallstone. Other factor includes the presence of concomitant pathology leading to colonic luminal narrowing, such as diverticulitis, inflammatory bowel disease (IBD), chronic constipation and neoplasm. Individuals who have undergone abdominal surgery, particularly those involving bowel anastomosis, are also at increased risk [10]. In the presented case, the patient had a 2.5-cm stone impacted in a healthy sigmoid colon. Although there is no evidence in the current literature that a prostatic cancer can be considered a factor, it is may be the cause of stone impaction.

Although surgery is considered the mainstay of treatment of gallstone ileus, the management of patients with sigmoid gallstone ileus should be tailored according to the patient’s clinical condition [8]. Moreover, age, comorbidities, BMI and overall clinical status are prospects with significant importance to be considered. Taking into account that this entity is more common in older patients with comorbidities and frailty, the management of sigmoid gallstone ileus has been evolving to less invasive approaches [8, 11]. Various approaches have been advocated including conservative, endoscopic, lithotripsy and surgery with varying success rate [8].

To our knowledge, only five cases have been reported in the literature demonstrating conservative management of colonic gallstone ileus, with four reported cases of a successful spontaneous dislodgment of the stone [8]. This noninvasive approach can be an acceptable option in selected patients, which include bowel rest, colonic lavage, cleansing enemas and watchful waiting for a spontaneous passage of the stone [8, 11]. Sigmoid obstruction can rarely resolve transanally; however, Farkas N. *et al.* reported an unprecedented manual transanal extraction of an impacted stone formally lodged in the sigmoid colon [12]. Once a conservative approach has been advocated as a management strategy, spontaneous dislodgment of the stone must be confirmed with CT scan or colonoscopy as symptomatic relief can sometimes be deceiving [13]. Prompt management of certain cases should not be delayed for a trial of conservative management, mainly when the patient is diagnosed with shock or signs of impending perforation, as treatment delay will most likely lead to severe complications [8, 13].

Many reports in the current literature demonstrated the use of transanal ileus tube for the management of mechanical colonic obstruction [14]. This approach can be helpful in relieving the obstruction, alleviating the patient’s symptoms and reducing the bowel edema. Furthermore, it can be used as a bridge to a semi elective surgery allowing for more time for perioperative management and also decreasing the risk of intraoperative complication such as perforation and peritoneal contamination. Transanal ileus tube has been used in cases of colonic gallstone ileus. It can decompress the colon proximal to the stone, which will reduce the inflammation, edema and aid in restoring the elasticity of the colonic wall, with subsequent dislodgment of the stone [15].

Previous reports of non-surgical management strategies such as endoscopic lithotripsy and endoscopic extraction of the colonic gallstone with a snare catheter, Talon grasping device or Roth Net retrieval basket have yielded varying results [10]. The current trend is towards primary endoscopic treatment, with varying success rates, as high as 14% in some series [8]. These techniques are limited by the availability of local expertise and are dependent on the size, composition of the impacted stone and the degree of luminal stenosis [11]. Although there has been reports of successful endoscopic management of an obstructing colonic gall stone as big as 4.5 cm, and as big as 6 cm when combined with lithotripsy; however, there are not enough data in the current literature that demonstrate a correlation between the size of the obstructing stone and the success of non-surgical management [11].

Surgical management of patients with colonic gall stone ileus is similar to certain extent to those with gallstone ileus occurring in the small intestines [8]. Although there are various surgical management strategies reported, enterolithotomy considered the main surgical option in absence of complications [12]. Farkas *et al.* used the appendectomy orifice to extract the stone by milking it to the cecum, rather than creating a colotomy in a diseased sigmoid colon [12]. Conversely, signs of perforation or ischemia warrant surgical resection. Sigmoid resection or Hartman’s procedure are feasible options [10]. Trephine colostomy is a less invasive technique that can overcome the conventional approach of stoma formation in high-risk patients who are not fit for general anaesthesia. It is associated with less operative time, limited skin incision and the ability to be performed under local or regional anaesthesia. However, this approach remains an unpopular option due to its technical difficulty, misidentification of the target organ and poor visualization of the operative field [10, 16]. The debate continues as to whether to perform a single stage surgery in which you perform enterolithotomy, along with cholecystectomy and closure of the fistula, or to perform a two-stage procedure with initial enterolithotomy to manage the obstruction followed by interval management of the fistula along with cholecystectomy [8]. By undertaking a single-stage surgery approach, the risk of recurrence and cholangitis are reduced. However, it is considered an extensive surgery that is associated with a prolonged operative time and an increased risk of postoperative morbidity and mortality [8]. On the other hand, several new series described a comparable mortality rate when comparing enterolithotomy alone with single-stage procedure. One of the recent reports, illustrated an improvement in the mortality rate, which is nuanced by advances in diagnostic modalities and improvement of the perioperative care. It has demonstrated a 7.32% mortality rate with single-stage surgery, and a 4.89% mortality rate with enterolithotomy alone, compared with previous reports of 16.9 and 11.7%, respectively [6, 17].

On the other hand, adopting two-stage surgery strategy can result in post-operative diarrhoea due to the passage of bile salt to the colon. This can be managed by ERCP and sphincterotomy [17]. This approach is still associated with lower mortality rate and allows for an interval elective definitive surgery, or no surgery at all, as spontaneous closure of a cholecystoenteric fistula may occur, particularly when the distal obstruction is relieved [10, 17].

The use of laparoscopy in the management of gallstone ileus has been previously described and was shown to have lower incidence of major complications and acceptable conversion rates of 11%. The current data, however, are limited to two case reports of successful management of colonic gallstone ileus [11, 17].

## CONCLUSION

Gallstone ileus remains a challenging case to manage with multiple factors playing role in choosing the best modality for treatment with either operative or non-operative approach. Non-operative management of sigmoid gallstone ileus is a feasible option in a clinically stable patient without signs of peritonitis, as spontaneous resolutions can occur. There is a need for a well-structured study for the best approach.

## Data Availability

The data underlying this article are available in the article and in its online supplementary material.
